# To Go Forward, We Must Look Back: The Importance of Evolutionary Psychology for Understanding Modern Politics

**DOI:** 10.1177/1474704918764506

**Published:** 2018-06-18

**Authors:** Rose McDermott, Peter K. Hatemi

**Affiliations:** 1 Brown University, AK, USA; 2 Pennsylvania State University

**Keywords:** evolution, politics, technology, power, institutions, social media

## Abstract

As new waves of populism arise and cause disruption around the globe, there is both great interest in attempting to explain the origin of this dynamic as well as a need to ameliorate its potentially destructive impact. Perhaps the greatest signal of seismic change is the global dismantling of American institutional control of the postwar world following the election of Donald Trump in the United States. In the wake of such dramatic changes, it may seem odd to turn to evolutionary psychology which looks deeply into the past to try to understand current events, but, in fact, modern technology has dramatically changed the shape of political communication in just such a way as to make politics more personal once again, increasing the need to understand and interpret modern politics through an evolutionary lens. In fact, current modern political turmoils demonstrate how important evolutionary themes are and how critical they remain to understand how current forms of populism tape into older tribal sentiments and drives. Modern technology allows for a form of interpretative politics that no longer need to be mediated by political institutions or larger social structures, including enduring ones such as marriage. Indeed, in any ways, as we have technologically advanced, we have also regressed to more immediate, emotional, and personal forms of political communication. And it is only in understanding the nature of that personal political psychology that we can begin to grapple seriously with the challenges of today, including the consequences of global populism.

As new waves of populism arise and cause disruption around the globe, there is both great interest in attempting to explain the origin of this dynamic as well as a need to ameliorate its potentially destructive impact. Even entrenched and established modern Western democracies have not been immune to the forces of public opinion, causing havoc among the public and the elite. Examples of this phenomena abound, including leaders trying to deal with the consequences of the Brexit vote in the United Kingdom; Marine Le Pen’s strong showing and Emmanuel Macron’s historic presidential victory in France, which swept aside a generation of leaders and sent French politics into upheaval; and the inability of Angela Merkel, who appeared unassailable just a few years ago, to form a government in Germany.

Perhaps the greatest signal of seismic change is the global dismantling of American institutional control of the postwar world following the election of Donald Trump in the United States. In the wake of such dramatic changes, it may seem odd to turn to evolutionary psychology which looks deeply into the past to try to understand current events, but, in fact, modern technology has dramatically changed the shape of political communication in just such a way as to make politics more personal once again, increasing the need to understand and interpret modern politics through an evolutionary lens. In fact, current modern political turmoils demonstrate how important evolutionary themes are and how critical they remain to understand how current forms of populism tape into older tribal sentiments and drives. Modern technology allows for a form of interpretative politics that no longer need to be mediated by political institutions or larger social structures, including enduring ones such as marriage. Indeed, in any ways, as we have technologically advanced, we have also regressed to more immediate, emotional, and personal forms of political communication. And it is only in understanding the nature of that personal political psychology that we can begin to grapple seriously with the challenges of today, including the consequences of global populism.

It should not be surprising to readers of this journal that the fundamental topics which lie at the intersection of evolution and politics are similar. These issues surround those enduring challenges which have confronted humans over long periods of time as they have sought to organize into collectives for the benefits of cooperation, the protection of collective welfare, and to provide the comfort of community, all while striving to avoid predation, exploitation, and starvation. These issues may appear with different manifestations across time and geography, so that the particular context will look different in a hunter-gather group than in 19th-century Britain, and different still in the modern American context, but topics of concern typically devolve to conflicts around resource allocation, in-group defense and out-group discrimination, and attempt to regulate sex and reproduction (Hatemi & McDermott, 2012).

In the modern American context, clearly evident in the 2016 presidential election, those underlying topics appeared manifest in arguments over benefits like access to health care, the status of transgender individuals and a woman’s right to choose, how to view out-groups such as immigrants, and how we defend ourselves in the war against terrorism. Significantly, every one of these issues emerged as a contention around the nature, rights and restrictions involved in various aspects of social status (whether those involved race, gender, ethnicity, sexuality, or some other demographic characteristic), reproduction, security, or the allocation and provision of basic needs. Modern sociologists point to the way in which Trump was able to exploit the disaffection of often poor, largely rural White males in particular to produce his electoral victory (Hochschild, 2016), but the rise of tribal politics of course extends far beyond the United States and has effects much more widespread than the election of Trump. While it may be accurate to blame the exacerbation of identity politics on the Democratic party, and certainly Trump and the Republican elites have played on and exacerbated such divisions, the social and political fractures which underlie the rise of populism in the United States and Europe find their roots far beneath Trump. In reality, identity politics simply represents the overt political manifestation of underlying human needs for affiliation, belonging, and community. These needs are not restricted to any particular political party or ideology but rather emerge from the fundamentally social aspect of human nature. In this way, we are not at all distinct from other social animals who need each other to survive and thrive in a hostile world characterized by limited resources. Within the social psychological tradition, social identity theory by Tajfel (1982) pointed to the benefits in self-esteem and in-group cohesion as well as the costs in terms of out-group discrimination that followed in the wake of such identification with similar others. But of course, the motivational drive to affiliate with similar others does not result from the group itself but rather resides in intrinsic human psychological benefits which derive from a sense of belonging. And the origin and impetus of these psychological traits and mechanisms is precisely where evolutionary psychology is uniquely poised to inform our understanding of the nature of political divides and offer useful suggestions moving forward.

Indeed, research over the last decade or so across many disciplines has shown how evolutionary theory can prove critical to understanding how we arrived at this current state of political polarization and division and to offer insights into how we might move toward greater cooperation and less conflict. A deep and broad literature has focused on in-group and out-group dynamics and morality. Other work has shown that when people feel under threat, their political opinions move more to the right, and they become more authoritarian as an evolutionary ingrained reaction to protect oneself (Haidt, 2008; Lewis & Bates, 2013). More recently, some have focused on physical features of why some candidates appeal to our primordial needs of group belonging (Bamshad et al., 2003; Klofstad, 2016), while others have explored more social reasons which motivate group belonging. Hogg, Hohman, and Rivera (2008) delineate three of these. The first is sociometer theory (Leary & Baumeister, 2000) which argues that people join groups for reasons of self-esteem; this model provides similar justification and benefits to that offered by older models of social identity theory. The second derives from terror management theory (Greenberg, Pyszczynski, & Solomon, 1986), which locates the motivation for group membership in individuals’ desire to avoid thinking about their own death and thus try to manage their fear around this reality through group affiliation. From this perspective, groups validate individual worldviews, including, for example, religious beliefs, which people find comforting. Finally, uncertainty-identity models (Hogg, 2007) speak to people’s need to reduce uncertainty through the security offered by groups which help define attitudes, norms, and roles. Group identity helps solve the functional epistemic problem of predicting what other people will do by provided established practices and preferences; they also provide the validation noted as significant by terror management theory.

Note, however, that this perspective focuses on more cognitive aspects of identity, while contestation of identity often occurs along other dimensions as well including social, relational, and constitutive (Abdelal, Herrera, Johnston, & McDermott, 2006). This remains significant because many of the current political divides occur along these other fractures. The rise of populism and populist discourse illustrates divisions along all these lines. This is where the growing and targeted research stream focusing on the role of evolutionary psychology and its related models becomes so directly relevant in seeking to understand the renewed rise in populism. Populism is a type of political movement highly dependent upon charismatic leaders who seek to mobilize mass constituencies through rhetoric that takes on an oppositional persona, in which the leader is simply one of the people, pitted against the system and the “elite.” Populism can take on either left and right positions; populists are diverse in most all respects, with few shared characteristics with the exception of a deeply held and shared antiestablishment viewpoint.

From traditional academic perspectives of political analysis, understanding populism presents a challenge. Indeed, there is little consistent theoretical or categorical consensus around which to elucidate populism. This is perhaps where evolutionary theory can offer comparative advantage. For example, some have explicated the evolutionary foundation of dominance to explain the rise of populist leaders (Henrich, 2015; Kakkar & Sivanathan, 2017). McAdams (2018) suggests Trump’s appeal lies in his ability and willingness to channel “primal dominance…. Like the alpha male of a chimpanzee colony, Trump leads (and inspires) through intimidation, bluster, and threat….” (p. 1). This argument builds upon Wrangham and Peterson’s (1996) and De Waal and Waal’s (2007) groundbreaking works surrounding chimpanzee politics and the connections between apes and humans across various forms of human behavior including aggression. Human proclivity for social hierarchy, display behavior, and the psychological need for dominant leaders traces back millions of years through human evolution. Leader traits, including dominance, can be seen within the larger schema of charisma, which is believed to have “evolved as a credible signal of a person’s ability to solve a coordination challenge requiring urgent collective action from group members” (Grabo, Spisak, & van Vugt, 2017: 417). As noted in the work mentioned above, the urgency and importance of successfully accomplishing coordination tasks are increasingly relevant during times of uncertainty, whether economic or physical safety. Kakkar and Sivanathan (2017), for example, find uncertainty leads to feelings of loss of personal control, which affects individuals’ psychological desire to for dominant leaders.

In addition, recent models drawing on an evolutionary theory of leadership emergence suggest that dominance and prestige present dual routes to leadership (Cheng & Tracy, 2014). For example, recent work shows the ways in which leaders can emerge from particular constellations of pride and personality. Cheng, Tracy, and Henrich (2010) note that:

…hubristic and authentic pride, respectively, may be part of the affective-motivational suite of psychological adaptations underpinning the status-obtaining strategies of dominance and prestige…. Moreover, the two facets of pride are part of a larger suite of distinctive psychological traits uniquely associated with dominance or prestige. Specifically, dominance is positively associated with traits such as narcissism, aggression, and disagreeableness, whereas prestige is positively associated with traits such as genuine self-esteem, agreeableness, conscientiousness, achievement, advice-giving, and prosociality. (p. 334)

Other research has focused not so much on leader traits but more specifically on conditions that elicit states of uncertainty and threat. Perhaps one of the strongest human drives outside of mating and meeting basic survival goals is the need to be part of the group. Humans are highly responsive to pain, and group ostracism elicits the same mechanisms of pain and stress as physical pain (Kross, Berman, Mischel, Smith, & Wager, 2011). From an evolutionary perspective, being rejected from your social group translates into a loss of resources, status, access to mates, physical protection, and ultimately survival. The same risks exist today, though to a much lesser extent as they have been mitigated by laws and social protections, at least somewhat. Nevertheless, isolation can still produce all kinds of risk, including risks to one’s health (Holt-Lunstad, Smith, Baker, Harris, & Stephenson, 2015). In other words, the most basic physical and psychological processes require social support for optimal functioning.

Populist movements rely on inflammatory rhetoric to create a tribal “us versus them” condition—this type of environment instigates neural mechanisms from the evolutionary desire to be part of the group (Kurzban & Leary, 2001). When the public believes there is a threat to their group, there is a natural instinct to become more groupish and defend the group (Duckitt, 2006). Recent Populist leaders in Europe (e.g., Denmark, France, Hungary, Sweden, and the United Kingdom) and the United States all focus on the potential threat posed by immigrants to elicit this defensive instinct and to draw support for often unrelated public policies. Perhaps no better example is Trump’s presidential announcement on June 16, 2015:

When Mexico sends its people, they’re not sending the best. They’re not sending you, they’re sending people that have lots of problems and they’re bringing those problems…. They’re bringing drugs, they’re bringing crime. They’re rapists…

Such messages, despite being xenophobic and bigoted, trigger underlying drives for self-defense of one’s community, further moving people toward populist leaders. For example, messages that counter the group’s narrative often trigger a neural stress response. That is, individuals who might question group values, or take in information dissonant to their in-group, risk social exclusion. In an evolutionary sense, this would risk death, and although such attacks may no longer risk literal death, threats to one’s group can feel existential in nature and trigger the same psychological mechanisms and defenses which have evolved to motivate people to seek out the protection of the group when such sanctuary often made a life or death difference. This is one reason why individuals’ first and often lasting response is strongly negative when they perceive their values to be under attack, and why simply more education or the “right” information does little to change one’s opinion (Kahan et al., 2011; Westen, Blagov, Harenski, Kilts, & Hamann, 2006).

Threat and fear trigger responses designed to protect the group; they provide a psychological cue which serve to engage self and group defense. As noted above in the discussion of terror management theory, a great deal of evidence shows that when mortality salience is triggered, the public will increasingly endorse the use of violence to protect themselves (Landau, Solomon, Pyszczynski, & Greenberg, 2007). The threats need not be real or immediate to evoke this response. And in today’s world of advanced democracies, they rarely are. Symbols, images, and words now represent the greatest threats most peoples in democracies ever face, but as a result, these threats to worldviews and values have come to fully substitute for threats that were once much more severe, personal, and physical (Landau et al., 2007). Symbols become a surrogate for immortality and, as such, come to provide an element of psychological protection. As a result, if someone’s idea is attacked, that person feels threatened. Simply being exposed to the image of person from a different political, religious, ethnic, sexual, or social group can thus trigger a threat response—as do their ideas. Populism simply provides an extension of identity politics, and in an evolutionary sense, all individuals want their group to be the one on top, whether they recognize or are willing to acknowledge that reality (Nisbet, Cooper, & Garrett, 2015).

As can be seen, much work in this area has already been done, but there is much more to do. Here, we point to two specific areas where greater attention and research might help provide some productive avenues for understanding the deepening nature of the current political divide and working to achieve greater cooperation. These involve the role of technological innovation in facilitating communication, and the role of institutions in structuring and enforcing constraints on individual action.

## How Technology Flipped the Power Structure for Political Discourse

On any given day, anyone who watches or reads about what comes out of the White House will witness a fantastic and increasing conflation of politics with entertainment, a never-ending theatre of veritable Greek tragedy. This should not be surprising, given President Trump’s background in reality television, and, of course, politics has always incorporated an element of showmanship. The current administration, however, has seemingly eliminated any division between the two industries. Recent reports even claim that Trump sees everyday as an episode that should leave people hanging on for more (Price, 2018), and of course the media colludes with him by giving him all the attention he clearly craves.

In a century from now, assuming that the human species still exists, we may look back and find that perhaps no technological advance has had a greater influence on democracy than the advent of social media. And, as a result, in an odd twist, we are now politically closer to that small bands of humans that we came from than at any other time since the preindustrialization era. Politics in advanced societies, until very recently, relied on institutions, elites, and media for information, control of the message, and communication. This all changed. Now, more than even in our recent industrialized past, political discourse reverted back to local, tribal, and individually driven activities and behaviors because of the widespread and immediate communication facilitated by technological innovations such as the World Wide Web and smartphones. Individuals can now generate and drive memes and narratives en masse wholly independent of elite intermediation. If individuals or small groups of people, not much larger than early communities of humans, can interact, develop, and drive their own political discourse outside of elite narratives, interaction becomes less centralized, more democratic, and also much more chaotic and messy. Because of these technological innovations, a single person can have a direct conversation with millions of people, without any elite influence or regulation, and do so in real time, as if they were all in the same room. This is not to say that such forms of interaction are inherently better or worse than elite-driven discourse but rather points to the fact that less centralized forms of communication are now ubiquitous and come with certain predictable challenges. For example, when the Russian people threw off their oppressive and repressive authoritarian leaders and reverted to their earlier Russian heritage after the collapse of the Soviet Union in 1991, hyperdemocracy ruled for a brief period of time. While everyone felt like they could and should exert their voice, and less coercion was possible, nothing got done either because a visceral aversion to hierarchy in the wake of authoritarian rule meant a complete lack of the ability to facilitate the efficient separation of labor and division of tasks (Fish, 1996). As a result, as the economy faltered and their international status plummeted, Russians increasingly privileged order over freedom, and returned to a more authoritarian political structure, resulting in Putin’s resurgent popularity.

But the speed and pace of technological innovations has precipitated even more rapid transformations in the areas of communication and information than was possible in the early 1990s in Russia. Twitter and text messages have been used to organize political campaigns in Tehrir Square in Egypt to oust an entrenched dictator, to mobilize opposition to the government in the early days of the civil war in Syria, as well as more recent demonstrations against the government in Iran. Increasingly, easily taken and transmitted cell phone video is used to document all sorts of violations which are then used to generate outrage among supporters and activate behavioral responses to political and social transgressions. In this way, technology facilitates an extremely low-cost mechanism by which a single person can communicate with millions of others without interruption or mediation.

Political leaders such as President Trump have found that Twitter provides a means by which to engage in an end run around a main stream media, otherwise known as the fourth estate, whose critical role is enshrined in the first amendment to the Constitution under the right to freedom of speech, but the reality is that Twitter, like social media in all its forms, is a two-way street. It is very likely the founding fathers, it alive today, would be appalled that their goal of democracy was extended so far beyond the landed White men they were intent on enfranchising, but the reality is that the idea was bigger than their original creation and came to extend far beyond their wildest dreams. Ordinary citizens can also use Twitter, Facebook, Instagram, and other forms of social media to similarly circumvent not only the media but the president or other leaders and elites as well, in any way or form they choose. The costs of organization have never been lower and the ease of organization has never been higher.

Because social media is cheap, individuals no longer need money to be noticed. This low-cost exposure for all kinds of ideas that might have previously never made it into the wider public discourse supports the many ways in which more and more aspects of political life have become local; as observers decry the siloed echo chambers of our current media culture, many individuals can take advantage of this opportunity to circumvent elite structures. This broad-based democratization can offer a voice to many but also, in union, poses a serious and sustained threat to the existing political and social order. Without making any normative claims one way or another, it is important to recognize the dramatic shift back to a more personal and local politics which these technological innovations have precipitated.

Thus, many people no longer need to anthropomorphize state institutions and structures in order to think about their relationship to government, politics, and their rights and responsibilities with regard to others. Individuals can quickly and easily revert to more natural and automatic forms of personal politics without having to worry about, or work through, larger political or governmental structures. They can get their messages and views out immediate and directly to millions through Twitter and Facebook in ways that not even governments were able to do a quarter century ago. This means that in thinking about political goals and outcomes, individuals no longer have to try to figure out how to make their ideas operate through abstract institutions far away; rather, they can talk to the whole world like it is in their living room because for all intents and political purposes, it is.

However, the challenge from an evolutionary perspective in adapting personal relationships and processes to the whole world is that the scale of such interactions are much larger than those in which the psychology designed to negotiate politics developed. Individuals who may be trying to communicate with a relatively small group can instead reach hundreds of thousands, if not millions of others, resulting in many unintended consequences and unforeseen and unpredictable feedback loops. So while institutions previously regulated which ideas could reach larger audiences, the reach of social media, while colossal, is not necessarily as personal as people experience. Many of those who receive a message may not agree and may in fact be provoked by ideas they consider threatening, even if they emanate from half way across the world. And it is not clear that people properly calibrate to that larger scale of connectivity, even if feels like a more natural direct form of communication than the institutionally mediated form of information that dominated the recent past.

## The Role of Institutions in Regulating Individual Action

The ruling institutions are coming under increasing attack in the newest wave of global populist uprising. Many elites express deep concern about the incipient destruction of the global neoliberal economic and political order which has been dominated by the United States since the end of the Second World War. Values endorsing capitalism and democracy helped shape institutions such as the United Nations, the World Bank, the World Health Organization, the World Trade Organization, the Import–Export Bank, the G-8 partnership, and the Bretton Woods system. Such institutions were enforced globally for the past three quarters of a century, clearly the blink of an eye in evolutionary terms, through the unprecedented power and reach of the U.S. military. Many would claim that this period of American global leadership has led to similarly unprecedented levels of prosperity, if certainly not unbroken peace. It has also led to heightened global levels of economic, sexual, and racial inequality, a not unlikely source of much populist opposition to these elite structures.

But, as noted above, ubiquitous and powerful technological innovations have allowed individuals to transcend the elite discourse and structures which had previously controlled the majority of lives across human history. Rather than having to work through the organizations, institutions, and corporations which had been structured by the elite for their own benefit, the new structures, perhaps best epitomized in the business world by the start-up culture prevalent in Silicon Valley, are more human centered, focusing on the power of social capital and information technology than on industrial infrastructure or material production.

Until quite recently, the bigger organizations, institutions, and corporations were, the more power they could exert over others’ behavior. But technology has helped precipitate an almost complete flip in that power structure, such that individuals can now exert the kind of power once reserved only for large nations. The best example of this, of course, is a terrorist organization like Islamic State of Iraq and the Levant (ISIS) that can wield total annihilation within a failed state structure; if such groups were able to obtain nuclear, chemical, or biological weapons, they could indeed succeed in accomplishing a level of destruction that even very large state militaries were not able to achieve in the past. As existing political and social institutions become increasingly discredited because they seem to serve only the elite, one person who can reach millions through social media can exert a disproportionate influence in framing the social narratives the guide popular mobilization.

Institutions, organizations, and laws have worked to impose order, predictability, and other constraints on individuals, at a certain cost of freedom. Under the guise of some kinds of equality, such as equality of opportunity, other kinds of equality, most notably those related to outcome, are readily sacrificed. Yet without the governing capacity of such large organizations, social order can dissipate, and the reversion to a preference for a strong leader readily and easily emerges, along with the concomitant downstream consequences that can arise under authoritarian kinds of control.

## Implications for Understanding the Modern Political World

In this way, individuals can rely on, use, and benefit from an innate psychology that naturally and automatically interprets all politics as local and personal, albeit through the mechanisms of technology and social media. Yet the scale of this technological reach is more akin to large-scale institutions, organizations, and corporations than it is to a small village. And so the challenges of maintaining cooperation within such large groups remain daunting, and thus the sources of, and motivations for, social fractures which allow people to divide into increasingly narrow slivers of identity politics become obvious. Reverting to such trial identities, including the clan-based ones so prevalent across the desert band of Northern Africa, the Middle East, and Central Asia or the increasingly fractured demographic alliances that govern current American politics, allows people to maintain cohesion within their much larger state collectives. Larger groups can split apart simply because of coordination challenges. Smaller groups are easier to keep together. Identity provides a simple and often quickly visually obvious way to separate others into friends and foes, allies, and enemies. No matter how imperfect the divide, such categories provide the sense of social community and security which individuals crave in order to feel a sense of belonging and security. This can be true even if the community is illusory, the benefits limited (if predictable), and the costs relatively high. Because at root, politics is not just about providing norms for how I should live my life; they are primarily designed to give everyone the sense of legitimacy and authority which they feel gives them license to tell other people how they should live their lives.

As with many other aspects of human life, evolutionary drives and motives instantiated in specific psychological adaptations allow processes designed for one environment and situation to be hijacked by another than manages to mimic or simulate the relevant cues to activation. Mass publics may want to benefit from the order imposed by authoritarian rule, but such preferences developed in environments where cheater detection was easier because communities were smaller, and leaders who did not benefit their constituencies, or disproportionately took resources for themselves, were decapitated by their followers (Boehm, 1999; Cosmides, Tooby, Fiddick, & Bryant, 2005). When such processes have to be adapted to a modern world where national communities can include millions of people from quite different backgrounds such as the United States, it can prove much more difficult to detect true exploitation, particularly if everyone knows their enemies can lie about such tendencies for their own benefit or to properly punish transgressions directly. Laws and institutions intervene and prevent many natural inclinations, such a father who might want to kill a new husband of his children’s mother. But in the absence of institutions which have achieved consensus legitimacy, the reversion to tribal identities becomes necessary for sheer protection on many levels. The drive for this can become particularly urgent when institutions are seen to privilege certain groups at the expense of others, thus losing widespread legitimacy.

Current challenges facing the planet are numerous and urgent, from global problems like climate change that will require unprecedented collective action in order to mitigate, to resource scarcity in the face of overpopulation, to the overwhelming consequences of mass migration resulting from these forces. As people compete for basic resources in a world of increasing deprivation, conflicts will become more numerous, fierce, and inescapable. Answers to these questions and challenges are not obvious. But what is obvious is that for any solution to work, it must take into account the natural human tendencies, drives, desires, and incentives of a human psychology powerfully shaped by evolutionary forces. Without such a conscious consideration in the design and implementation of future institutions and organizations, failure becomes as inevitable as it will be predicable. Our hope is that this reflection and the essays included in this special collection can enhance our understanding of the critically intertwined interaction between evolutionary psychology and political process and structures and advance our prospects for achieving a more just, prosperous and peaceful world for everyone.

## The Special Issue

This article does not simply constitute a call to arms to encourage more research to be conducted but also serves as introduction to the current special issue. This special issue is devoted to the theme of trying to understand, explain, and illustrate how critical models and theories drawn from evolutionary psychology are critical to understanding modern political behavior and discourse. The author in this special issue demonstrates a variety of ways in which incorporating an evolutionary perspective into an analysis of modern issues generates new insights into the enduring challenges humans confront in striving to construct and maintain social life in groups, which is, in fact, the fundamental job and role of politics. The topics raised in this special issue cover a wide range of topics. Some of this work examines the effects of various biological factors on electoral preferences, including a piece which examines the role of voice pitch in voting across different kinds of electoral contests (Banai et al.) as well as one which explores the effect of facial dominance on voting preferences (Lausen et al.). Additional research investigates the role of sexual disgust in political preferences in the 2016 American election (Liberman et al.). The influence of sex and gender extends beyond voting in additional articles which analyze various aspects of the male warrior hypothesis, including a piece which looks at the difference in responses to male versus female terrorist bombings (Linder), and another which examines the assessment of competence across gender in the American presidential election in 2016 (Grabo & Van Vugt). Additional topics receiving attention include work on the problem of labor recruitment in offensive versus defensive battles (Lopez), the influence of corruption and hypocrisy on mechanisms of social control (Eldakar et al.), and the evolutionary origins of system justification beliefs (Jost & Sapolsky). All of these articles unite in their application of evolutionary models, and the hypotheses inspired by this approach and these theories, to modern concerns and problems.
